# Complete mitochondrial genomes provide current refined phylogenomic hypotheses for relationships among ten *Hirundo* species

**DOI:** 10.1080/23802359.2020.1790999

**Published:** 2020-07-20

**Authors:** Javan K. Carter, Peter Innes, April M. Goebl, Benjamin Johnson, Matthew Gebert, Ziv Attia, Zachariah Gabani, Ruiqi Li, Tina Melie, Chiara Dart, Ali Mares, Chrisopher Greidanus, Jaime Paterson, Brianna Wall, Gabriela Cortese, Kevin Thirouin, Gabrielle Glime, Joseph Rutten, Cameron Poyd, Erin Post, Brianna Wall, Ahmed A. Elhadi, Katherine Feldmann, August Danz, Thomas Blanchard, Samantha Amato, Stephan Reinert, Cloe S. Pogoda, Elizabeth S. C. Scordato, Amanda K. Hund, Rebecca J. Safran, Nolan C. Kane

**Affiliations:** a Department of Ecology and Evolutionary Biology, University of Colorado, Boulder, CO, USA; b Department of Biological Science, California State Polytechnic University, Pomona, CA, USA; c Department of Ecology, Evolution, and Behavior, University of Minnesota, Minneapolis, MN, USA

**Keywords:** *Hirundo*, phylogenomic, mitogenome, phylogenetic

## Abstract

*Hirundo* is the most species-rich genus of the passerine swallow family (Hirundinidae) and has a cosmopolitan distribution. Here we report the complete, annotated mitochondrial genomes for 25 individuals from 10 of the 14 extant *Hirundo* species; these include representatives from four subspecies of the barn swallow, *H. rustica*. Mitogenomes were conserved in size, ranging from 18,500 to 18,700 base pairs. They all contained 13 protein-coding regions, 22 tRNAs, a control region, and large and small ribosomal subunits. Phylogenetic analysis resolved most of the relationships between the studied species and subspecies which were largely consistent with previously published trees. Several new relationships were observed within the phylogeny that could have only been discovered with the increased amount of genetic material. This study represents the largest *Hirundo* mitochondrial phylogeny to date, and could serve as a vital tool for other studies focusing on the evolution of the *Hirundo* genus.

## Introduction

The genus *Hirundo* comprises 14 species of swallows and has a cosmopolitan distribution spanning every continent except Antarctica. Of these species, the barn swallow (*H. rustica*) is the most widespread, breeding throughout Eurasia and North America, and overwintering across the Southern Hemisphere (Brown and Brown 1999). Eleven of the *Hirundo* species reside primarily in Africa, and three others inhabit the Pacific. Members of *Hirundo* build mud-cup nests in a variety of habitats (e.g. cliffs, anthills, human dwellings, bridges) and are highly adapted to aerial feeding. Among members of the genus *Hirundo*, variation in melanin-based plumage color is thought to be a prominent aspect of phenotypic differences. In addition to differences in plumage color among members of the genus *Hirundo*, length of outer tail streamers and body size tend to be helpful aspects of phenotype for identifying species (Dor et al. 2010). Differences in these character traits are often minimal, which presents challenges to taxonomic efforts (Sheldon et al. 2005; Dor et al. 2010).

Dor et al. published a species tree of all members of the genus *Hirundo* using one nuclear gene and six mitochondrial DNA genes with the purpose of understanding lineage diversification and phylogeographic relationships within this clade (Dor et al. 2010). This was significant as this was the only phylogeny of the *Hirundo* genus using more than 1000 base pairs of genetic material at the time. An earlier paper by (Sheldon et al. 2005) presented the entire swallow family, *Hirundinidae*, and used even fewer genes, thus a lower base pairs count, for its phylogeny. The ability to sequence and assemble entire mitogenomes is now more feasible than in the past due to reduced financial costs and improved computational accessibility; re-evaluating the phylogeny using more genetic data is now a more achievable task.

In the present study, we revisited the evolutionary relationships among members of the genus *Hirundo* by assembling complete mitochondrial genomes of 10 species. Genomes were assembled *de novo* from Illumina shotgun sequence data. These mitochondrial genomes offer greater resolution and support for some aspects of the previous Dor et al. (2010) *Hirundo* mtDNA gene tree and also offer insight to a new topology.

## Materials and methods

### Sample collections

Samples were collected from both museum breast tissues and field collection blood samples as depicted in Table 1.

**Table 1.** Complete collection of samples used in analysis.

Sample collection Data table							
Tissue sample ID #	Species	Specimen type	DNA concentration (ng/µl)	DNA extraction date	Collection location: (lat/long or location)	Museum/Museum ID	GeneBank accession #
RS-44	*Delichon urbicum*	T	293.362	3/29/19	South Africa	LSUMNS/B-14047	MN853682
RS-45	*Delichon urbicum*	T	141.935	3/29/19	South Africa	LSUMNS/B-14048	MN824431
RS-52	*Delichon urbicum*	T	155.650	3/29/19	Greece	LSUMNS/B-25370	MN832895
RS-53	*Delichon urbicum*	T	214.133	3/29/19	Greece	LSUMNS/B-25371	MT427586
RS-37	*Hirundo aethiopica*	T	91.684	3/29/19	Cameroon, Ouest Province	LSUMNS/B-27162	MN844887
RS-35	*Hirundo aethiopica*	T	137.131	3/29/19	Cameroon, Ouest Province	LSUMNS/B-27160	MN850676
RS-42	*Hirundo albigularis*	T	92.275	3/29/19	South Africa	UNMM/177227	MN829450
RS-39	*Hirundo angolensis*	T	351.931	3/29/19	Uganda, South Buganda Province	LSUMNS/B-25384	MN849177
RS-40	*Hirundo atrocaerulea*	T	298.491	3/29/19	Africa, Malawi	FIELD/467951	MT442038
RS-21	*Hirundo dimidiata*	T	312.766	3/29/19	South Africa, Cape Province	LSUMNS/B-14124	MN832869
RS-27	*Hirundo dimidiata*	T	178.112	3/29/19	South Africa	LSUMNS/B-14132	MT471263
RS-12	*Hirundo neoxena*	T	474.249	3/29/19	Australia, Victoria	LSUMNS/B-23632	MN844886
RS-20	*Hirundo neoxena*	T	91.772	3/29/19	Australia, South Australia	LSUMNS/B-14187	MN848412
RS-41	*Hirundo nigrita*	T	79.570	3/29/19	Africa, Democratic Republic Congo	FIELD/473429	MN832899
RS-43	*Hirundo nigrita*	T	104.301	3/29/19	Africa, Equatorial Guinea	YPM ORN/100623	MN849307
L-324	*Hirundo rustica tytleri*	B	100.318	2017	Russia Long: 52.021259 Lat: 106.590942	NA	MN843972
L-48	*Hirundo rustica rustica*	B	32.796	2017	Russia Long: 57.558842 Lat: 62.662777	NA	MN829439
L-242025	*Hirundo rustica transitiva*	B	80.819	2017	Israel Long: 32.9282 Lat: 35.5407	NA	MN954681
L-242007	*Hirundo rustica transitiva*	B	67.697	2017	Israel Long: 32.9282 Lat: 35.5407	NA	MN840495
L-1607	*Hirundo rustica savignii*	B	73.561	2017	Egypt Long: 31.407243 Lat: 31.785907	NA	MN830163
RS-1	*Hirundo smithii*	T	62.538	3/25/19	South Africa, Transvaal	LSUMNS/B-14115	MN853142
RS-5	*Hirundo smithii*	T	130.802	3/29/19	Ghana, Northern Region	LSUMNS/B-39509	MN629932
RS-6	*Hirundo smithii*	T	423.404	3/29/19	South Africa, Transvaal	LSUMNS/B-14119	MN849178
RS-29	*Hirundo tahitica*	T	89.936	3/29/19	Papua New Guinea	LSUMNS/B-25389	MN833781
RS-33	*Hirundo tahitica*	T	98.726	3/29/19	Malaysia, Sadah	LSUMNS/B-61614	MN849306

Tissue samples ID: arbitrary units used as identification during the DNA extraction, sequencing, and genomic analysis stage. Specimen type: T: tissue sample stored in liquid nitrogen collected from museum; B: Blood sample stored in Blood Lysis Buffer (Hoelze). DNA concentrations: DNA concentrations were calculated using Biotek Synergy HT Multi-Detection Microplate reader (Held and Buehrer 2003). Collection location: This is displayed either with a general location (i.e. Country, county/city) or latitudinal and longitudinal coordinates collected during tissue/ blood extraction. Museum/museum catalog number: LSUMNS: Louisiana State University Museum of Natural Science; FIELD: Field museum of natural history in Chicago Illinois; UNMM: University of New Mexico Museum; YPM: Yale Peabody Museum. GenBank Accession: This is a representation of the current administered accession numbers.

#### DNA extraction and sequencing

Genomic DNA was extracted using a QIAamp DNA Blood Mini kit, following instructed blood or tissue protocol. Genomic libraries were then prepared using Nextera^®^ XT DNA library prep kits (Illumina^®^), and each sample was barcoded using unique dual index adapters Nextera^®^ i5 and i7 by the University of Colorado’s BioFrontiers Institute Next-Generation Sequencing Facility in Boulder Colorado. Samples that passed quality control were processed for paired end 150 base pair reads on the Illumina Novaseq S4 sequencer (800 raw data/lane) at Novogene^®^.

#### Mitochondrial genome assembly

The whole genome data were aligned to the *Hirundo rustica gutturalis* reference mitogenome (GenBank accession KP148840.1) with Samtools function *bwa-mem* using default settings (Li 2013). Only the reads that aligned to the mitochondria were retained for use in downstream assembly (this subsetting of the data improved assembly performance). Reads were trimmed of adapters and low-quality reads using Trimmomatic v0.39 (Bolger et al., 2014)) with the following parameters: Illuminaclip: NexteraPE-PE.fa:2:20:10 Leading:20 Trailing:20 Sliding window:4:15 Minlen:100. *De novo* assembly of trimmed reads into scaffolds was performed with SPAdes v3.11.1 (Bankevich et al., 2012). The relative position, order, and orientation of scaffolds were determined by comparison with available *H. rustica rustica* and *H. rustica erythrogaster* reference genomes available on GenBank (GenBank accessions KP148840.1, KX398931.1). When multiple contigs represented the same genomic region, contig selection was based on maintaining approximate consistency in read coverage across contigs. Properly ordered contigs were then combined by trimming overlapping sequences. Gaps between scaffolds were filled by tiling from raw or trimmed reads.

#### Mapping and error correction

Assembled genomes were then aligned to reference *Hirundo* mitochondrial genomes (GenBank accessions KP148840.1, KX398931.1) with Zpicture, which allowed visualization of structural differences between the reference and our mitochondrial genomes (Ovcharenko et al., 2004). Assembled genomes were also compared according to relatedness for structural agreement following the work of Dor et al. (2010). Samtools tView was then used to identify possible SNPs (single nucleotide polymorphisms) or Indels (insertions and/or deletions) in the genome; modifications were made if mapped reads supported these assembly errors/variants (Li 2011). Completed draft assemblies were compared using Multiple Alignment using Fast Fourier Transform (MAFFT) ver. 7 (Katoh and Standley 2013). Large discrepancies in assemblies based on alignment to neighboring or same species were taken into account when making changes to the draft genome sequences.

### Annotation

Genomic features were annotated using MITOS and MITOS2 (Bernt et al., 2013). Accuracy of these annotations was checked by comparison to annotations from Mitofish (Iwasaki et al., 2013) and tRNAscan-SE 2.0 (Lowe and Eddy 1997). Annotations were further verified using BLAST to compare the nucleotide and translated protein sequences of our mitogenomes against the same reference mitogenomes as used in the alignment step (GenBank accessions KP148840.1, KX398931.1). Sequence and annotation information was entered in NCBI’s Sequin 15.50 software for final submission to GenBank. In some cases, Sequin identified errors in assembly (e.g. premature stop codons), which were then fixed via the methods described above.

#### Phylogenetic analysis

A maximum likelihood phylogenetic hypothesis was established using sequence data from the annotated whole mitochondrial genomes. The taxa list consists of 26 individuals from 10 species: *Delichon urbicum (n* = 4), *Hirundo atrocaerulea* (*n* = 1), *Hirundo dimidiata* (*n* = 2), *Hirundo tahitica* (*n* = 2), *Hirundo neoxena* (*n* = 2), *Hirundo albigularis* (*n* = 1), *Hirundo nigrita* (*n* = 2), *Hirundo smithii* (*n = 3*), *Hirundo angolensis* (*n* = 1), *Hirundo aethiopica* (*n* = 2), *Hirundo rustica tytleri* (*n* = 1), *Hirundo rustica rustica* (*n* = 1), *Hirundo rustica transitiva* (*n = 2*), and *Hirundo rustica savignii* (*n* = 1). Sequences were aligned in MAFFT (v7) in a CLUSTAL format (Katoh and Standley 2013). RAxML (v.8.2.10) was used to infer the phylogenetic hypothesis (Stamatakis 2014). To analyze node support 1000 Bootstrap replicates were used in a GTR GAMMA maximum likelihood search. The tree was rooted to *Delichon urbicum*, per the previous *Hirundinidae* mtDNA phylogeny, which shows *D. urbicum* being the closest relative apart from *Ptyonoprogne fuligula* (Sheldon et al., 2005).

## Results and discussion

### Genome annotation

Mitochondrial genome content across birds has been studied intensively, and has been found to generally include the following features, which were present in all of our assemblies: 13 protein-coding genes (ND1, ND2, ND3, ND4, ND4l, ND5, ND6, COI, COII, COIII, ATPase6, ATPase8, and Cytb), 22 tRNAs, and the large and small rRNA of the ribosome (Boore, 1999). We found incomplete stop codons for COXIII and NAD4, which is a common feature of avian mitogenomes (Anmarkrud and Lifjeld 2017). Post-transcriptional polyadenylation of RNA is a likely completion mechanism of these stop codons, however, studies of transcriptomic data will be necessary to verify this hypothesis. The mitochondrial genomes also exhibit characteristic compactness, with a maximum separation of 40 base pairs between genes. The vast majority of genes were encoded on the + strand. Of the 37 identified mitochondrial genes, only nine were encoded on the − strand, grouped into two operons and two standalone genes. Additionally, the genes were ordered identically across all sampled species, including the outgroup *D. urbicum*. The length of the highly variable rrnS and rrnL genes were relatively consistent across species, with rrnS measuring ∼1000 base pairs across species, and rrnL measuring ∼1600 base pairs.

### Phylogenetic comparison

In recreating the *Hirundo* genus phylogeny, several differences can be observed between the previous Dor et al. phylogeny and the current complete mitochondrial genome phylogeny topologies.

**Figure 1.**
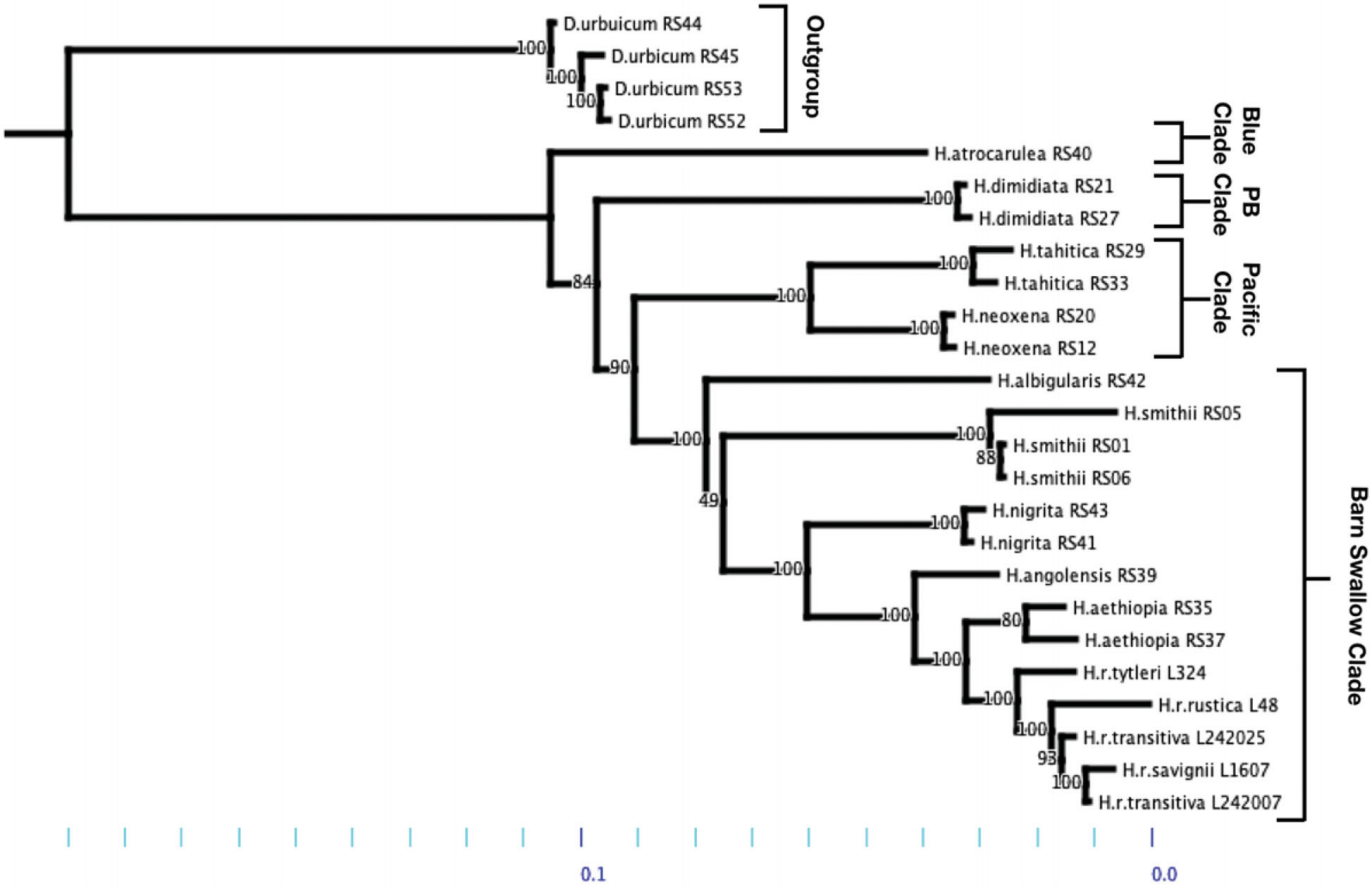
Maximum-likelihood phylogenetic hypothesis using GTR-GAMMA model parameter. Taxa (*n* = 26) rooted to Delicum urbicum. Outgroup: *Delicum urbicum*. Blue Clade: *Hirundo atrocarulea*. PB Clade (Pearl-Breasted Clade): *Hirundo dimidiata*. Pacific Clade: *Hirundo tahitica* & *Hirundo neoxena*. Barn Swallow Clade: *Hirundo albigularis, Hirundo smithii, Hirundo nigrita, Hirundo angolensis, Hirundo aethiopia, Hirundo rustica tytleri, Hirundo rustica rustica, Hirundo rustica transitiva*, and *Hirundo rustica savignii*.

We found two main differences between our *Hirundo* phylogeny and those preceding it (Sheldon et al., 2005; Dor et al., 2010); both differences are within the ‘Barn Swallow’ clade (figure 1). First, we found an unresolved polytomy within the ‘Barn Swallow clade associated with the *H. albigularis*, *H. smithii*, and *H. nigrita* taxons: the hypothesis of *H. nigrita, H. angolensis, H. aethiopica, H. r. tytleri, H. r. rustica, H. r. transitiva, H. r. savignii* being nested within *H. smithii*, as previously suggested, was not well supported (bootstrap support = 49/100)(figure1). This indicates uncertainty in the position of *H. albigularis* relative to *H. nigrita* and *H. smithii*. Geographically, *H. albigularis* and *H. smithii* species boundaries overlap in the southern regions of Africa, making a moderate amount of gene flow a potential causal factor for the polytomy, apart from the lack of time since divergence (Maddison and Knowles 2006) (figure 1). Second, while *H. angolensis* was originally nested within the *H. rustica* clade, our data show the opposite pattern of *H. rustica* along with *H. aethiopia* placed within the *H. angolensis* clade (figure 1).

Our findings also clarify a previously unresolved node connecting the ‘Blue Swallow’ and ‘Pearl-Breasted Swallow’ clades with the joint ‘Pacific Swallow’ and ‘Barn Swallow Clades’ (Dor et al., 2010). We found that the latter two clades are sister to the ‘Pearl-Breasted Swallow’ clade (bootstrap support = 84/100). The previous phylogeny (Dor et al., 2010) had suggested this relationship; however, a low bootstrap value of 54/100 prevented a definitive conclusion.

In addition to an updated tree topology, our whole mitogenome data also provide greater support for branch length values. Higher support within branch length is generally associated with more genetic data that agrees with the topology patterns and high levels of congruency (Wiens et al., 2008).

It is interesting that the whole mitogenomes presented here have clarified certain aspects of the *Hirundo* phylogeny while at the same time introducing an unresolved polytomy. An exact topology remains elusive even at the level of whole mitochondrial genomes. We suspect this is attributable to how closely relate these species are. Despite the benefits mitogenomes offer for phylogenetic studies, it is important to remember mitochondrial genes are linked and ultimately represent a single, matrilineal locus. We anticipate additional nuclear genomic markers will be of use in resolving all nodes of the *Hirundo* phylogeny. Future phylogenomic studies of *Hirundo* will investigate whether the nuclear gene tree agrees with the mtDNA tree presented here. The potential ILS associated with the polytomy between *H. albigularis*, *H. smithii*, and the *H. nigrita* clade will also be addressed using a phylonetwork approach during the nuclear genome analysis.

## Data Availability

The authors confirm that the data supporting the findings of this study are available within the article [accession information for GenBank in Table 1]. No supplementary material was needed.
